# Activation of CO and CO_2_ on homonuclear boron bonds of fullerene-like BN cages: first principles study

**DOI:** 10.1038/srep17460

**Published:** 2015-12-02

**Authors:** S. Sinthika, E. Mathan Kumar, V. J. Surya, Y. Kawazoe, Noejung Park, K. Iyakutti, Ranjit Thapa

**Affiliations:** 1 SRM Research Institute, SRM University, Kattankulathur, Tamil Nadu, 603203, India; 2 New Industry Creation Hatchery Center (NICHe), Tohoku University, Sendai, Japan; 3 Thermophysics Institute, Siberian Branch, Russian Academy of Sciences, Russia; 4 Center for Multidimensional Carbon Materials, Institute for Basic Science (IBS), Ulsan 689-798, Republic of Korea; 5 Department of Physics and Nanotechnology, SRM University, Kattankulathur-603203.

## Abstract

Using density functional theory we investigate the electronic and atomic structure of fullerene-like boron nitride cage structures. The pentagonal ring leads to the formation of homonuclear bonds. The homonuclear bonds are also found in other BN structures having pentagon line defect. The calculated thermodynamics and vibrational spectra indicated that, among various stable configurations of BN-60 cages, the higher number of homonuclear N-N bonds and lower B:N ratio can result in the more stable structure. The homonuclear bonds bestow the system with salient catalytic properties that can be tuned by modifying the B atom bonding environment. We show that homonuclear B-B (B2) bonds can anchor both oxygen and CO molecules making the cage to be potential candidates as catalyst for CO oxidation via Langmuir–Hinshelwood (LH) mechanism. Moreover, the B-B-B (B3) bonds are reactive enough to capture, activate and hydrogenate CO_2_ molecules to formic acid. The observed trend in reactivity, viz B3 > B2 > B1 is explained in terms of the position of the boron defect state relative to the Fermi level.

The prospect of utilizing non-metal materials for the adsorption and catalytic conversion of toxic environmental gases, as an alternative for the present-day precious metal catalyst is gaining interest, owing to its lower price as well as a better durablility^1^,^2^,^3^,^4^,^5^,^6^. Among metal-free adsorbents, carbon based nanostructures, such as C_60_, carbon nanotube (CNT) and graphene have received much attention^7^,^8^,^9^. Similar interest is directed to, BN analogue: it was discussed that with modified electronic structures it can also lead to promising materials for gas capturing and catalytic convertors^10^,^11^,^12^,^13^. The BN based monolayer and nanotube structures have been quite widely studied experimentally as well as theoretically^14^,^15^. It is noteworthy that, a recent experimental study has demonstrated the possibility of systematically converting a graphene sheet to a hexagonal BN sheet via a chemical route^16^. Combining the chemical route with the lithography technique it is possible to produce uniform boron nitride structures without disrupting the structural integrity. Also the carbon based template can be used to synthesize the BN structures^17^. Inspired by these experiments, in the present work, we explore properties of fullerene-like BN cages, hereafter named as BN-60, which may be obtained as a result of atom by atom substitution of C_60_ or by direct synthesis. The important point is that the network of pentagonal rings in BN-60 will lead to homonuclear bonds^18^. The BN cages, free of the homonuclear bonds, are made up of square and hexagon rings as discussed in previous literature^19^,^20^,^21^. However, pentagon–octagon–pentagon line defects are found in the BN sheets, nanoribbons, and single-walled BN nanotubes and are consequence of the existence of homonuclear bonds^22^. Under boron rich environment the large possibility of formation of frustrated B-B homonuclear bond has been reported^23^. Also the pentagons with homonuclear bond form at the tip of the h-BN nanotube^24^. In the present work, we found that the homonuclear bonds have decent reactivity, which is distinctly different from the conventional BN structures. We are particularly interested in the catalytic performance of homonuclear bonds for CO oxidation and CO_2_ conversion.

The oxidation of CO is an important prerequisite for mitigating toxic CO gas. On a catalyst surface, CO oxidation follows Langmuir–Hinshelwood (LH) mechanism and the Eley–Rideal (ER) mechanism. LH mechanism involves the coadsorption of reactants onto the catalytic surface, followed by a surface reaction to form the products. ER mechanism, on the other hand, involves the direct reaction of a gaseous reactant with a chemisorbed one. Nitrogen-doped carbon nanotubes possess the ability to effectively catalyze the CO oxidation with activation energies ranging from 0.477 to 0.619 eV. A less negative charge on the dopant N atom is correlated with a higher activity for CO oxidation^25^. Iron embedded graphene also proved to be a potential material for CO oxidation with activation energy of 0.58 eV^26^. Graphene doped with Cu results in electronic resonance among the electronic states of the reactants and the Cu atom, leading to higher reactivity for oxidizing CO. The process proceeds first via an LH mechanism with barriers of 0.25 eV and 0.54 eV followed by ER reaction without energy barrier^27^. *Zhao et al.* have investigated theoretically the possibility of CO oxidation on a Si embedded graphene surface and attributed to the charge transfer from the embedded Si atom to the 2π* orbital of O_2_. The process proceeds first via LH mechanism with a barrier of 0.48 eV followed by ER mechanism^28^. Fe encapsulated boron nitride cage has good CO to CO_2_ conversion capabilities with an activation energy of 0.5 eV^29^. The choice of dopants significantly alters the CO oxidation mechanism and hence the activity of boron nitride monolayer. This was demonstrated in our previous work employing carbon and oxygen as dopants, wherein the O dopant enabled chemisorption of CO, while C doped h-BN monolayer has lesser tendency to adsorb CO^30^. O doping results in a larger bond length of a neighboring B atom, it’s out of plane displacement and less positive charge, synergistically contributing to stronger CO adsorption.

Metal-organic frameworks, Carbon and BN nanostructures, such as CNT and BN nanotube (BNNT)^31^,^32^,^33^, have also been tested for CO_2_ capture and storage. As the weak binding of CO_2_ on such inert surfaces is a demerit, various methods to activate the surface have been tested. *Suchitra et al.* reported that Boron doped C_60_ (BC_59_) fullerene does not adsorb CO_2_ molecule effectively but 1e^−^ charged BC_59_ can strongly adsorb CO_2_ with binding energy of −0.66 eV^31^. *Huang et al.* proposed about the remarkable CO_2_ capturing ability of armchair graphene nanoribbons with dangling bond defect, the adsorption energy is about −0.31 eV^34^. *Sun et al.* provided a route to increase the activity of a pure BN sheet to adsorb CO_2_ by applying the electric field. By applying 1.36 eV of electric field the adsorption energy can be increased to −0.84 eV^35^. *Gao et al.* demonstrated that single Ca atom anchored on C_60_ can adsorb CO_2_ with higher binding energy compared to pristine C_60_^36^. *Shao et al.* proposed the increase of chemical activity of BNNT by the substitutional doping of Al atom in-place of B site. The CO_2_ binding energy varies with tube diameter and is in the range of about −0.03 to −5.08 eV^37^.

In the present work, the BN analogues of C_60_, which possess the frustrated homonuclear bonds because of pentagons, are investigated in the perspective of aforementioned CO oxidation and CO_2_ conversion catalyst. The stability of the cages has been analyzed and discussed in detail. It has been discoursed how the pentagonal rings in the structure will generate the homonuclear B-B, B-B-B, N-N and N-N-N bonds. Throughout this work, we designate B1 notation for a single B atom surrounded entirely by nitrogen, B2 for two B atoms bonded together, making a B-B bond and B3 for two adjacent B-B bonds merged to form a B-B-B bond for simplicity. Similarly for N sites we consider the similar notation. The binding affinity of the different stable BN-60 cages considering B1, B2 and B3 sites to capture CO/CO_2_/O_2_ molecules has been estimated. The role of the sites (B1, B2 and B3) on the CO oxidation and CO_2_ conversion are analyzed in detail using first principles approach.

## Results and Discussions

### Construction of BN-60 cages

Here we first outline the scheme adopted for the replacement of carbon atoms in C_60_ cage, containing 12 pentagonal and 20 hexagonal rings with B and N atoms to construct the BN-60 cages. In general, there are many ways to construct the BN-60 cages containing different distribution of B and N atoms, B/N ratio and homonuclear B and N bonds. In this work we made three different classes of BN-60 cages, considering 1. Boron rich, 2. Nitrogen rich and 3. stoichiometric B:N environment. To make the BN-60 cages with these environments, our approach is to make homonuclear B and N bonds first, taking into account pentagonal rings, and then following some rules. Initially, replacement of carbon atoms is done on pentagonal rings such that each pentagonal ring has one homonuclear bond of either type (B2 or N2). In fact, it is evident from recent experiments that the presence of homonuclear bonds on pentagonal rings of boron nitride structures are inevitable^20^. Another important consideration is that we restrict the homonuclear bonds to a maximum of three B (B3) and three N (N3) atoms. So to make B rich cages, all pentagonal rings are filled by B2 bonds and each B2 is surrounded by three or four homonuclear B2 bonds with one carbon atom separating them. This constraint prevents the formation of homonuclear N2, B3 or N3 configurations. The remaining carbon atoms in both hexagonal and pentagonal rings are replaced by B and N atoms alternatively, to avoid the N2 bonds. To incorporate B3 bonds in the boron rich condition, two or three B2 configurations should be surrounded by two or three B2 bonds.

The N rich BN-60 cages are constructed in a similar fashion incorporating N2 bonds instead of B2 bonds. In order to make stoichiometric B:N type BN-60 cages, the 12 pentagonal rings should be equally shared by both B2 and N2 bonds (6 B2 and 6 N2). Arrange these B2 and N2 bonds by alternative or continuous way first so that the number of B2 and N2 bonds is same and now depending on the surrounding homonuclear bonds, rest of the carbon atoms in both hexagonal and pentagonal rings are replaced by both B and N atoms. The steps for constructing stoichiometric *1*-B30N30 type cage with 5 B2, 2 B3 & 5 N2, 2 B3 bonds is demonstrated in the Fig. S1 of the supplementary information (SI). This approach would allow us to consider the effects of homonuclear bonds that are always likely to occur on pentagonal rings of BN nanostructures.

### Stability of BN-60 cages

In order to analyze the stability of different BN-60 structures, the formation enthalpy (F.E) per atom has been estimated using the following equation,


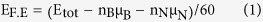


where, E_tot_ is the total energy of the different type of homonuclear bonded BN-60 structure. n_B_ is the number of boron atoms replaced the carbon atoms; μ_B_ is the chemical potential of the boron atom (reference structure: α-rhombohedral phase of bulk boron); n_N_ is the number of nitrogen atoms replaced the carbon atoms; μ_N_ is the chemical potential of the nitrogen atom (reference structure: N_2_ gas molecule). Higher negative values of formation enthalpy for BN-60 cages, as obtained from equation (1), indicate better stability of the system.

**Figure 1:**
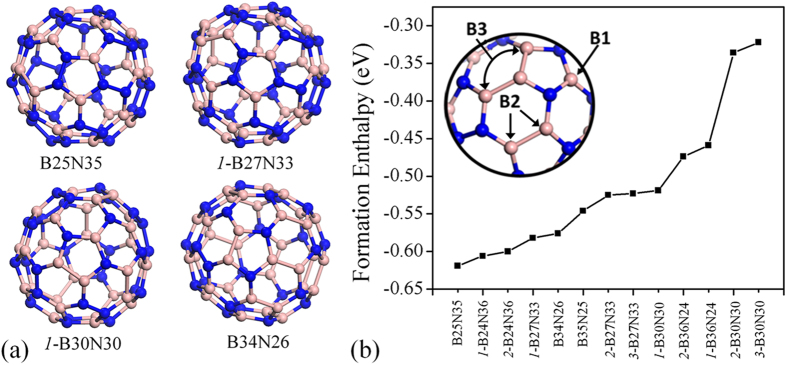
Stability of BN-60 cages. (**a**) Relaxed structure of BN-60 cages with different B:N ratio and homonuclear bonds. (**b**) Formation enthalpy per atom of various BN-60 cages. Blue and pink sphere denoted the N and B atoms.

In Table 1, the formation enthalpy values for different type of homonuclear bonded BN-60 cages are summarized. In every case the E_F.E_ is negative which clearly indicates the stability of the systems. The formation enthalpy value is plotted against the configuration of the system and shown in Fig. 1b and it helps to obtain a clear idea about the relationship between the formation enthalpy and type of BN-60 cage. Among all, N rich systems show a higher negative value than others, hinting that N- rich cages are more favorable to be synthesized in normal pressure and temperature. It is also evident that higher number of B3 bonds compared to the B2 bonds leads to lower stability of the systems, which can be easily understood comparing the formation enthalpy of three different types of B30N30 cages (see Table 1). Based on the formation enthalpy and considering homonuclear B bonds, four BN-60 cages (one B rich, two N rich and one stoichiometric B:N cases) are selected for further calculation and are shaded in grey in Table 1. Geometry optimization for the different type of BN-60 cages was performed and the optimized structure is shown in Fig. 1a and Fig. S2. The B rich or more B bonds in the system leads structural distortion and it is visible in Fig. S2 and Fig. 1a, so it may act as a good medium for gas adsorption. We estimated the density of phonon states (DOPS) for all the BN-60 cages. No negative frequency states because of structural instability have been found. Here only DOPS of four structures are shown in Fig. S3 (B25N35, *1*-B27N33, *1*-B30N30, B34N26). This indicates that all the structures are highly stable. The DOPS for all the other structures are not shown here.

### Density of states (DOS)

**Figure 2:**
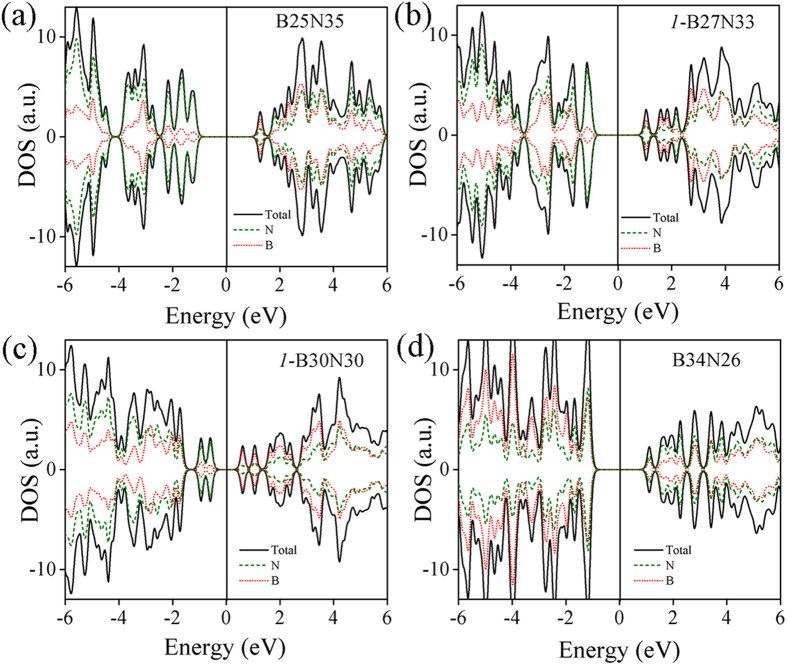
Electronic properties of BN-60 cages representing contribution of B and N atoms. Partial density of states (PDOS) of (**a**) B25N35 (b) *1*-B27N33 (**c**) *1*-B30N30 and (**d**) B34N26 cages. Black solid line, green dash line and red dotted lines represent the PDOS for total (B and N), only N and only B atoms. Fermi level is consider at 0 eV.

Next, we gain an understanding on the electronic properties of BN-60 cages. The densities of states (DOS) as a function of energy (eV) for four specific systems (B25N35, *1*-B27N33, *1*-B30N30, B34N26) are shown in Fig. 2. These results indicate that both B and N atoms in every structure contribute to the valence band maximum and conduction band minima. The contribution vary based on the B:N ratio in the BN-60 cages. For example in case of B25N35, N atoms mainly contribute valence bands whereas the B atoms contributed to the conduction band mostly. The B atoms‘ contribution to the valence bands increases in case of B34N26 cages. Also the B:N ratio and homonuclear B2, B3, N2 and N3 configuration play a major role for generating defect sates. In particular, the DOS for a *1-*B30N30 has defect states very near to the Fermi level because of the higher number of B3 and N3 configuration in the system. More understanding about the role of B1 B2 and B3 configuration is discussed in the next section.

### Adsorption of molecules

In order to test the catalytic capabilities of the BN-60 cages, we first study the adsorption of molecules, O_2_, CO and CO_2_. The high negative charge of nitrogen prevents the adsorption of any of the reactants on N1, N2 and N3 sites^30^,^38^. Table 2 shows the calculated adsorption energies of the molecules on the three different boron sites. It can be seen that the reactivity of boron atom strongly depends on its environment. As expected, B1 sites show no evidence of adsorbing any reactant molecules, rendering them unsuitable for catalytic applications. On B1 sites, the O_2_ adsorption energy (E_ad_(O_2_)) varies from 0.001/−0.057 eV (considering PBE/PBE-D functional) in *1*-B27N33 to −0.094/−0.198 eV in B25N35, CO adsorption energy (E_ad_(CO)) varies from 0.003 eV/−0.070 eV in B25N35 to 0.022 eV/−0.337 eV in B34N26 and CO_2_ adsorption energy (E_ad_(CO_2_)) is rather very small in all the cases. Interestingly, B2 atoms are more reactive, with E_ad_(O_2_) ranging from −2.492/−2.622 in *1*-B30N30 to −2.854/−2.988 in B25N35, E_ad_(CO) varying from 0.028 eV/−0.344 eV in B34N26 to −0.539/−0.652 eV in *1*-B27N33 and E_ad_(CO_2_) varying from 0.052/−0.079 in *1*-B27N33 to −0.195/−0.375 eV in B25N35. The optimized geometries of B27N33 after the adsorption of molecules are shown in Fig. S4. The high affinity toward O_2_ and CO combined with weak CO_2_ adsorption suggest that B2 sites of *BN-60 cages* can be efficient in catalyzing CO oxidation, which is discussed in detail later. The B3 sites strongly adsorb the O_2_, CO and CO_2_ molecules, with (E_ad_(O_2_)) ranging from −2.903/−3.043 eV in *1*-B27N33 to −3.470/−3.599 eV in *1*-B30N30, E_ad_(CO) ranging from −1.185/−1.294 in *1*-B27N33 to −1.678/−1.784 eV in B34N26 and E_ad_(CO_2_) varying from −0.196/−0.382 eV in *1*-B27N33 to −1.214/−1.361 eV in B34N26. The ability of B3 sites to anchor CO_2_ molecules is of particular interest as these systems can be employed as metal-free CO_2_ trapping agents to solve many environmental problems. On adsorption, the CO_2_ molecule is strongly activated as is evident from the bent geometry of the molecule (Fig. S4).

Bader charge analysis also confirms that the B3 sites are the highest reactive sites. The net charges are given in units of e, with a positive charge indicating a deficit of charge and a negative charge indicating a surplus of charge. The Bader charge analysis is performed, taking *1-*B27N33 as a representative case. Before adsorption, B1 has a net charge of +2.129. After the adsorption of CO, O_2_ and CO_2_ on *1*-B27N33, the net charge on B1 is +2.146, +2.145 and +2.135 respectively (given in Table 3), indicating that there is very little charge transfer to the incoming molecules. The net charges on the two atoms constituting B2 changes from +1.344, +1.441 before adsorption to +1.423, +1.577 on CO adsorption, +2.19, +2.16 after O_2_ adsorption and +1.319, +1.469 on CO_2_ adsorption and are given in Table 3. These values indicate that the B2 sites are able to donate electrons to the O_2_ and CO molecules whose net charges post adsorption are −1.545 and −0.268 respectively while the CO_2_ molecule is unaffected and retains its neutral charge. The three atoms constituting the B3 sites have charges of +1.25, +0.880 and +1.26 before the adsorption of any molecules which changes to +1.519, +0.949 and +1.411 on CO adsorption; +1.414, +1.378 and +2.16 on O_2_ adsorption and +1.405, +1.376 and +1.976 on CO_2_ adsorption. The charges on O_2_, CO and CO_2_ in this case are −1.54, −0.387 and −1.418 respectively. A point to note is that the charge transfer to the adsorbates is the largest when the boron sites before adsorption have less positive charge and hence more electron density. The central atom of the B3 site hence has the strongest ability to adsorb the incoming molecules.

**Figure 3:**
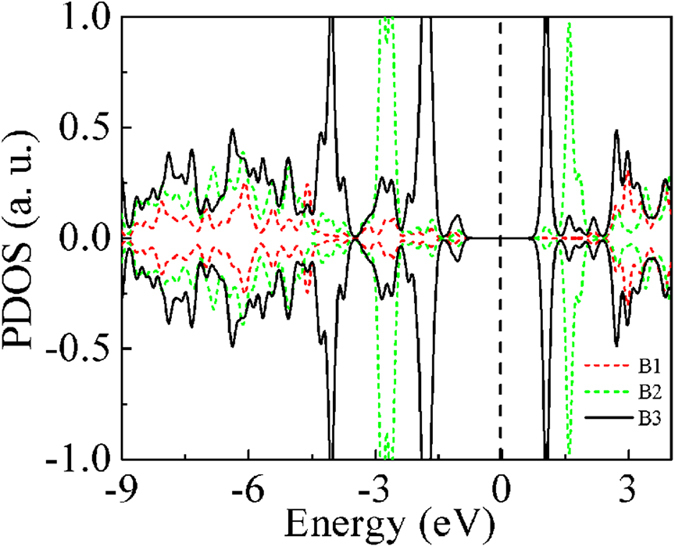
The partial density of states of the three different active boron sites of *1*-B27N33. Red dotted line indicates B1, green dashed line indicates B2 and black solid line indicates B3 PDOS. Fermi level is considered to lie at 0 eV.

In order to understand the origin of the observed trend in reactivity, *viz*., B3>B2>B1, we plot the partial density of states (PDOS) of B1, B2 and B3, taking *1*-B27N33 as an example as shown in Fig. 3. Inspection of the PDOS reveals that the occupied defect states of B3 sites lie near to the Fermi level (here normalized to lie at 0 eV), indicating their ability to easily transfer electrons to the reactant molecules. Also, the largest contribution to the B3 states comes from the central atom of B3.The defect valence band states of B2 sites are a little further away relative to the Fermi level, while the B1 states are far away from the Fermi level. Thus the position of the defect states because of the homonuclear configuration relative to the Fermi level governs the reactivity.

**Figure 4:**
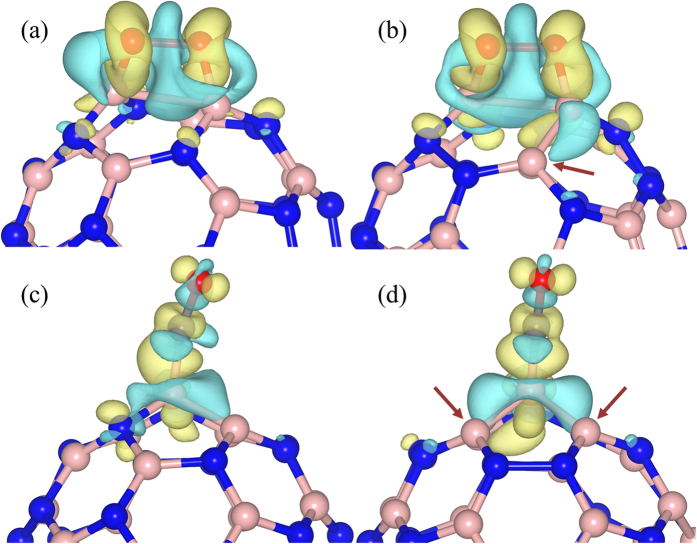
Charge density difference for O_2_ and CO adsorption. O_2_ anchored on (**a**) B2 site of B27N33, (**b**) B3 site of B27N33, and CO anchored on (**c**) B2 site of B27N33, (**d**) B3 site of B27N33. Blue and yellow lobes correspond to a depletion and accumulation of electronic charge, respectively. The isosurface value of 0.005 e/Bohr^3^ is considered for all the cases. The arrows indicate the boron atom/s in the active site not bonded with the adsorbate. Pink, blue, red, and gray balls indicate B, N, O, and C atoms, respectively.

To gain more insight on the reactivity of B2 site and B3 sites, we calculated the charge density difference (CDD) upon the CO and O_2_ adsorption on *1*-B27N33, as depicted in Fig. 4. The isovalue is set at 0.005 e/Bohr^3^. The yellow and blue lobes represent the charge accumulation and the charge depletion, respectively. The CDD plots well explain that the B3 sites are more active than the B2 sites in interacting with an incoming molecule. All the plots demonstrate that O_2_, CO and the surface undergo considerable charge redistribution on adsorption: the molecules acquire electrons from B27N33. The depletion of charge in the O-O and C-O bond regions of O_2_ and CO imply that the molecules are strongly bound to the surface, resulting in the elongation of the intramolecular bond. The charge depletion from both the atoms of the B2 sites upon the O_2_ adsorption can be clearly observed in the plot in Fig. 4(a). In addition, in the case of B3 sites (see Fig. 4(b)), the bond connecting the third B atom, which is indicated by an arrow and is not directly bonded with the incoming molecule, suffers from some amount of charge depletion, indicating that this site also plays a role in donating electrons to the incoming molecule. The observed trend of the higher reactivity of B3 site can be attributed to the overall charge donation feature of B3 atoms. The same observation is also found during CO adsorption. Even though CO molecule is attached to a single boron atom of either B2 or B3 (see Fig. 4(c,d)), the other boron atoms (indicated by arrows) constituting the homonuclear bonds also take part in electron donation to the incoming molecule, promoting the overall binding ability and hence B3 sites adsorb the strongest. This feature is also evident from Bader charge analysis, tabulated in Table 3, wherein all the boron atoms constituting the homonuclear bonds lose electronic charge on interacting with either CO or O_2_.

### Reactivity of homonuclear bond

**Figure 5:**
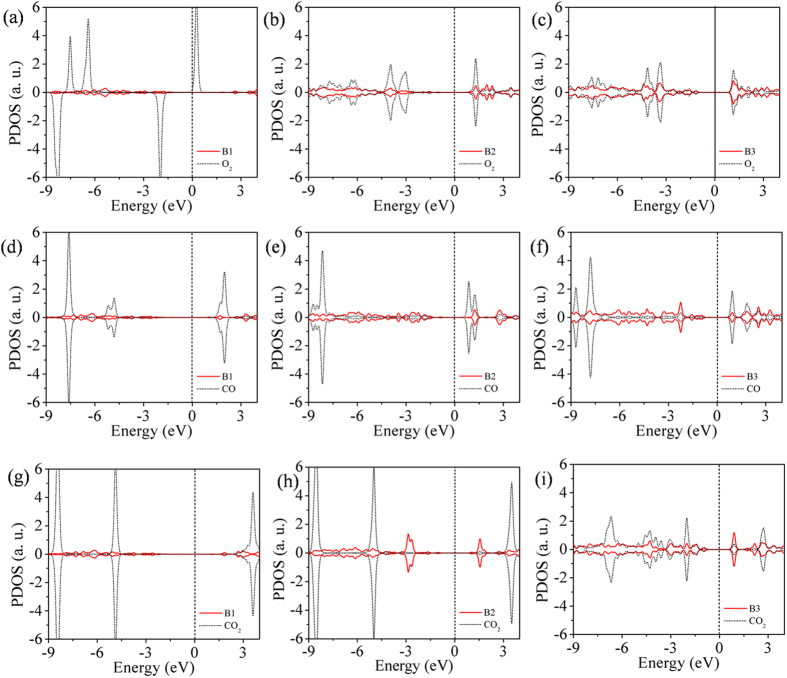
PDOS to explain the interaction behavior of the gas molecules with active boron sites. Partial density of states of (**a**–**c**) O_2_ on B1, B2 and B3 sites respectively; (**d**–**f**) CO on B1, B2 and B3 sites respectively; (**g**–**i**) CO_2_ on B1, B2 and B3 sites respectively of *1*-B27N33 after adsorption. Fermi level is consider at 0 eV.

The PDOS of the boron sites (B1, B2, B3) after the adsorption of molecules is shown in Fig. 5. It can be seen from Fig. 5a,d and g that there is no charge transfer from the B1 sites to any of the incoming adsorbate molecules. The molecular orbitals of O_2_, CO and CO_2_ retain their isolated characteristics, depicted in Fig. 5. Figure 5b,c show the PDOS of B2 and B3 sites and the O_2_ molecule upon adsorption. The spin-up π* (O_2_) states lie just above the Fermi level and can act as acceptor levels^39^. In fact, upon adsorption on B2 and B3 sites, these states become occupied, shift downward and hybridize with the p states of B2 and B3 atoms, explaining the higher interaction. Figure 5e,f demonstrate the DOS of B2, B3 and CO molecule after adsorption. The antibonding (π*) states of CO lie at around 2 eV above the Fermi level. As the valence states of B2 and B3 are closer to the LUMO of CO, the interaction of the CO molecule with B2 and B3 sites results in charge transfer to π* orbital, resulting in stronger adsorption.

The CO2 molecule does not interact with the B2 sites,as can be seen from Fig. 5h. The chemisorption of CO_2_ onto the B3 sites takes place in two steps: the bending of the CO_2_ molecule followed by its adsorption^40^,^41^. In order to understand this mechanism, we first performed a density of states analysis of an isolated bent CO_2_ molecule, keeping the bond lengths and bond angles same as the adsorbed configuration (see Fig. S5). The density of states clearly illustrates the splitting of the HOMO (1π) and LUMO (2π*) orbitals of the CO_2_ molecule into two states^42^. The split LUMO orbitals are named 2a and 2b, of which 2b can now readily accept electrons as it lies closer to the Fermi level. This can be understood by inspecting the DOS of B3 and CO_2_ after their interaction (Fig. 5i), wherein the 2b states become occupied, resulting in the weakening of C-O bond of CO_2_ and hence stronger adsorption. Based on the estimated results and analysis, we can conclude that for complete CO oxidation B2 sites are more suitable, and for CO_2_ capturing and conversion B3 sites are superior.

### CO oxidation and free energy profile

Now we investigate the mechanism by which CO oxidation occurs on B2 site of *1*-B27N33 and B25N35. This site is able to anchor both CO and O_2_ implying that the CO oxidation may follow the LH mechanism. The free energy profile is constructed by taking ΔG = ΔE – TΔS + ΔZPE, where ΔE is the total energy change obtained from DFT calculations, ΔS denotes the entropy change and ΔZPE is the change in the zero point energies. TS of free molecules are obtained from ref. 43, while TS of the adsorbates and ZPE of the free molecules and adsorbates are estimated from the DFT calculations considering vibrational frequencies of the molecules in the harmonic approximation, freezing the BN cage^44^. The ZPE correction is calculated as ZPE = ½∑_i_ћω_i_, where ћ is the reduced Planck’s constant and ω_i_ is the frequency of the i^th^ vibrational mode of the adsorbate molecule. The entropic term of the free energy is calculated from:


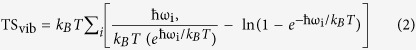


where 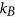 denotes the Boltzmann constant.

**Figure 6:**
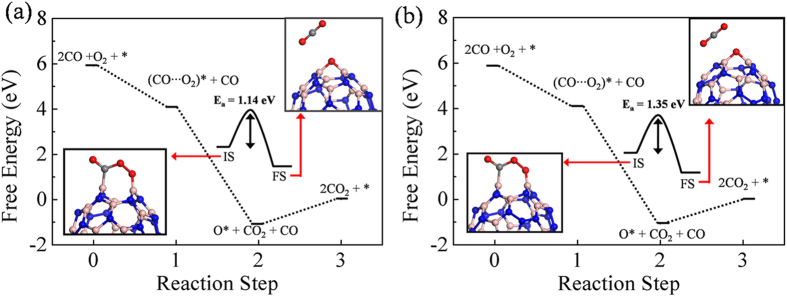
Free energy pathways of CO oxidation via LH mechanism on (**a**) *1*-B27N33 and (**b**) B25N35. The lower left insets show the co-adsorption of O_2_ and CO on B2 site, which is the initial state, while the upper right insets show the final state for the formation of first CO_2_. The initial state and final states are denoted as I.S. and F.S. E_a_ denoted the activation barrier. The *sign indicates the catalytic surface, and the adsorbed state of molecules and atoms are denoted with a *sign. Blue, pink, grey and red spheres denote the N, B, C and O atoms. The dotted lines are to guide the eye.

The images demonstrating the reaction steps of CO oxidation via LH mechanism is shown in Fig. S6. The initial step of LH mechanism is taken to be the one in which 2CO molecules and an O_2_ molecule are far from the surface and do not interact. The co-adsorption of CO and O_2_ on the B2 site is taken to be the next step and the optimized structure is shown in the inset of Fig. 6. The O_2_ molecule which was initially in the triplet state loses its magnetic moment upon adsorption. Detailed information on the changes in the spin state of molecular oxygen upon adsorption is explained in the supporting information and tabulated in Table S1. The desorption of first CO_2_ from the surface requires an activation energy (E_a_) of around 1.14 eV in *1*-B27N33 and 1.35 eV in B25N35. This step is hence the rate limiting step with the highest activation barrier. The overall reaction is exothermic (ΔG = −5.18 eV in *1*-B27N33 and −5.16 eV in B25N35) and the remaining O atom migrates toward the epoxy site. The O atom then readily reacts with another incoming CO molecule to generate the next CO_2_ molecule. This reaction requires that a thermodynamic barrier of 1.06 eV in *1*-B27N33 and 1.08 eV in B25N35 be surmounted. The calculated values of E_a_ and adsorption energies of reactants are used to estimate the Sabatier activities of CO oxidation over B27N33 and B25N35. The SA can be used as a measure of the ability of the catalyst to catalyze the process of CO oxidation. The first reaction step (R1 of SI) is taken as the one in which CO is adsorbed and in the next step the O2 molecules adsorb (R2 of SI) on neighboring active sites. This results in the formation of a (O_2_···CO)^*^ intermediate. The activation barrier for desorption of first CO_2_ from this intermediate plays a decisive role in the overall activity. Also we found that the very high binding strength of molecules on the surface influences the activity. The Sabatier activities of *1*-B27N33 and B25N35 are found to be −1.3 and −1.8 respectively. We have also calculated the SA of B30N30 and found it to be −0.61. The reaction rate is influenced by both the temperature and activation energies for CO oxidation. For instance, in the LH mechanism, after CO adsorption, O_2_ adsorbs in a neighboring site, forming a (O_2_···CO)^*^ intermediate. We have considered the removal of first CO_2_ from this intermediate to be the rate determining step (R3 in the SI), because of the high activation barrier. The Arrhenius equation is:


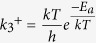


where 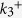 is the rate constant. Thus, the higher the temperature, the easier it is for the reactants to surmount the activation barrier^45^. In this work, the calculations of the rate constants, rate and the Sabatier activity are performed at a temperature of 273 K. At higher temperatures, the activation processes are expected to be faster. The detailed reaction steps and calculation procedure are outlined in the supplementary information. We have also compared the calculated Sabatier activities of the BN nanocages with a few other conventional catalysts available in the literature, and found that B30N30 cage performs good, showing the excellent activity (see Table S2 of supporting information).

### CO oxidation on boron nitride nanotube with defects

**Figure 7:**
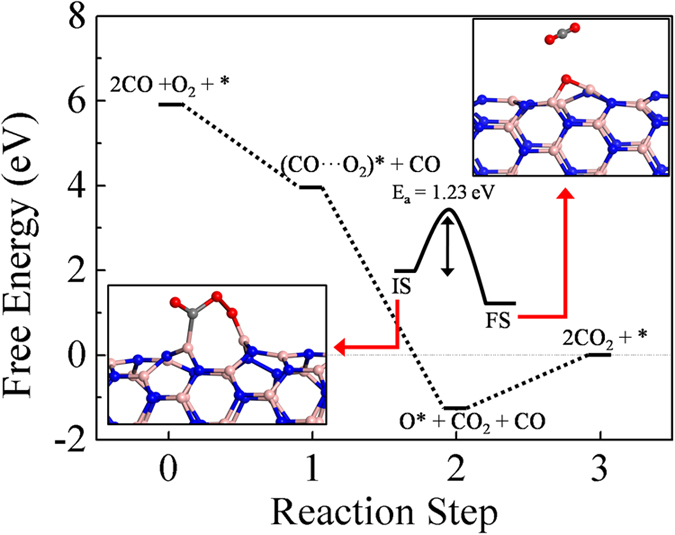
Free energy pathways of CO oxidation on SW-BNNT via LH mechanism. The initial and final states for the desorption of first CO_2_ are shown in the lower and upper insets respectively. E_a_ denotes the activation barrier. The sign conventions and atom colors are similar to that followed in Fig. 6. The dotted lines are to guide the eye.

The presence of similar kind of homonuclear B-B bonds have been observed in defective BN nanosheets and nanotubes. In particular the Stone-Wales defect (SW) in the BN based system has been studied theoretically^46^. Also, spectroscopic studies suggest the existence of such defects is more feasible in the boron nitride nanotubes^47^. A recent atomic resolution imaging study has also confirmed the presence of Stone-Wales like defects in boron nitride sheets^48^. To investigate the activation processes on B-B sites, we take an example of a SW defect on a BNNT (henceforth named as SW-BNNT). To model this system, we have chosen a (7, 0) supercell consisting of 42 BN formula units. The SW defect is formed by rotating a BN bond by 90^0^. The O_2_ molecule adsorbs at a B-B bond in the side-on fashion with adsorption energy of −2.97 eV, while CO adsorbs at the boron site of a B-B bond with adsorption energy of −0.08 eV, at a distance of 1.62 Ǻ from the surface. The adsorption energies of O_2_ and CO on SW-BNNT are stronger than those on a pristine BNNT. This is in agreement with previous results^49^. In order to compare the activity with the nanocages, we consider only the LH mechanism, wherein the CO molecule is adsorbed first followed by the adsorption of O_2_, to form a CO···O_2_ intermediate (see Fig. 7 for the entire energy profile). The removal of first CO_2_ requires an activation barrier of 1.23 eV, leaving behind an oxygen atom in the epoxy position. The high value of E_a_ can be justified based on the weak adsorption of CO molecule unto the surface, implying that O_2_ bond breaking is difficult. The reaction is exothermic by −5.23 eV. The reactive O atom then interacts with another CO molecule to form the second CO_2_ molecule. We note in passing that CO oxidation via ER mechanism is also probable and may take place requiring a smaller activation barrier as the O_2_ molecule adsorbs quite strongly in the side on fashion.

### CO_2_ conversion

**Figure 8:**
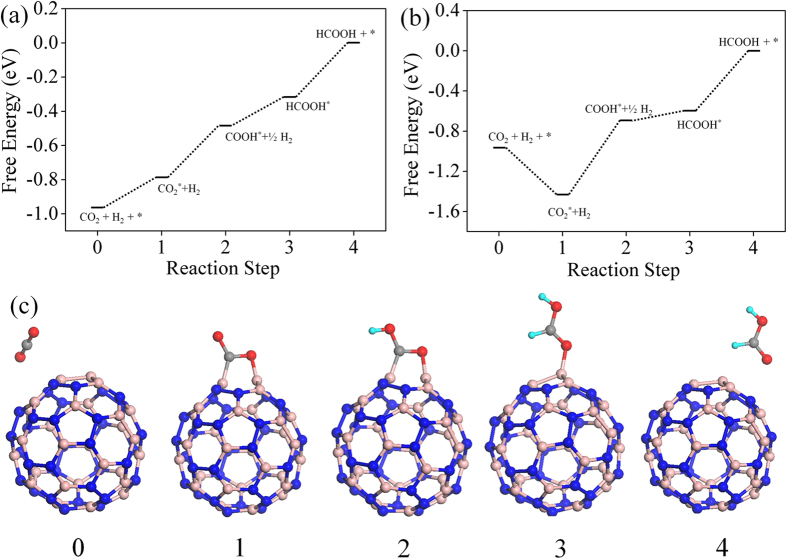
Free energy pathways of CO_2_ hydrogenation on (**a**) *1*-B27N33 and (**b**) B30N30. (**c**) Optimized structures for each reaction step considering *1*-B27N33 cage. The dotted lines are to guide the eye. The sky blue spheres represent hydrogen atom. The other colored spheres represent the same atoms as depicted in Fig. 6. The free hydrogen is not shown in the images for simplicity.

As mentioned earlier, the B3 sites are able to capture CO_2_ effectively in the BN cages. We examine here the possibility of effectively hydrogenating this activated CO_2_ into formic acid, which is widely used as a chemical fuel. We have taken two examples of BN cages, namely *1*-B27N33 and B30N30 to test the photocatalytic CO_2_ conversion capabilities via a COOH mediated mechanism^50^. The free energy profile for this process is shown in Fig. 8. We use similar convention to find the free energy change (ΔG) as discussed in previous section. Here it has been assumed that ‘
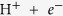
’ is in equilibrium with 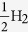, at pH = 0 and 0 V vs standard hydrogen electrode (SHE)^6^,^51^ Initially the CO_2_ molecule is considered to be far from the surface. The next step involves the adsorption of CO_2_ onto the B3 sites, which is endothermic in *1*-B27N33 by 0.18 eV (Fig. 8a). This is expected as the CO_2_ molecule does not bind very strongly to *1*-B27N33. But in the case of B30N30 (Fig. 8b), the adsorption of CO_2_ is stronger and hence the first step is exothermic (ΔG = −0.46 eV). The next step, which is the hydrogenation of the activated CO_2_ at its oxygen atom to form carboxyl (COOH), is uphill by 0.3 eV in *1*-B27N33 and 0.73 eV in B30N30. The third step wherein the carbon atom of COOH is attacked by a hydrogen atom to form adsorbed formic acid is mildly endothermic by 0.14 eV in *1*-B27N33 and endothermic by 0.10 eV in B30N30. Finally, the adsorbed product, HCOOH desorbs from the surface, with ΔG values being 0.34 eV and 0.60 eV in the case of *1*-B27N33 and B30N30 respectively. The low endothermicity of the reaction steps occurring on the B3 sites of *1*-B27N33 suggests an exciting possibility of hydrogenating CO_2_ at near-room temperatures.

## Conclusions

We design three different classes of BN-60 cages analogue to C_60_ cage considering 1. B rich, 2. N rich and 3. stoichiometric B:N environments. The pentagonal rings developed the homonuclear B and N bonds in the BN-60 structures. The formation enthalpy per atom for different configurations has been estimated and found to be in the range of −0.322 to −0.619 eV. N rich BN-60 cages are more favorable compared to other environments and the stability of BN-60 cage with more B2 configuration is higher compared to cages with B3 configuration. The DOPS confirms the good stability of the BN-60 cages. We found that the ability to anchor gas molecules follows B3 > B2 > B1. This trend has been explained considering position of the defect state relative to the Fermi level (B2 > B3). The stronger adsorption of the O_2_ and CO molecule on B2 and B3 sites is primarily because of charge transfer to π* orbital from the surface states. Only B3 sites can adsorb CO_2_ molecule, through bending of CO_2_ molecule, which results in the splitting of LUMO orbitals named 2a and 2b, of which 2b can readily accept electrons. The CO oxidation follows the Langmuir–Hinshelwood (LH) mechanism with Sabatier activity of −1.3 and −1.8 considering *1*-B27N33 and B25N35 cage. We found that B3 sites can efficiently convert the CO_2_ molecule to formic acid. Here we emphasize that the proposed science is a general understanding and will help us to proceed further for an efficient metal free catalyst.

## Methods

We performed the calculations using spin polarized density functional theory as implemented in the Vienna ab-initio simulation package (VASP)^52^. The generalized gradient approximation (GGA) was employed for the exchange and correlation effects at Perdew–Burke–Ernzerhof (PBE)^53^ and the potentials of the atoms were described by the projected augmented wave (PAW)^54^ method. For long-range van der Waals attraction the Grimme’s method (DFT-D2) was used with PBE functional (denoted as PBE-D)^55^. It was found that plane wave cut-off energy of 450 eV was sufficient to get well-converged results. Each BN60 cage was placed in a cubic supercell of size 15 Å, to avoid interactions between periodically repeating images which are at about 9 Å distances. Brillouin zone integration was performed at the Γ point only. All the structures were optimized until the total energy converged to less than 10^−5^ eV per atom and the maximum force converged to lower than 0.001 eVÅ^−1^. Density functional perturbation theory (DFPT) has been used to calculate the density of phonon states (DOPS)^56^. The nudged elastic band (NEB) method was employed to estimate the barrier energy^57^.

## Additional Information

**How to cite this article**: Sinthika, S. *et al.* Activation of CO and CO_2_ on homonuclear boron bonds of fullerene-like BN cages: first principles study. *Sci. Rep.* **5**, 17460; doi: 10.1038/srep17460 (2015).

## Supplementary Material

Supplementary Information

## Figures and Tables

**Table 1:** Number of homonuclear bonds and formation enthalpies per atom of different BN-60 configurations used in this study.

System		B-B	B-B-B	N-N	N-N-N	F.E (eV)
B24N36	*1*- B24N36	0	0	12	3	−0.606
	*2-*B24N36	0	0	18	0	−0.600
B25N35		1	0	12	2	−0.619
B27N33	*1*- B27N33	2	1	13	0	−0.582
	*2*- B27N33	1	2	14	0	−0.525
	*3*- B27N33	1	2	14	0	−0.523
B30N30	*1-*B30N30	5	2	5	2	−0.519
	*2-*B30N30	4	4	4	4	−0.336
	*3-*B30N30	11	1	11	1	−0.322
B34N26		12	1	2	0	−0.576
B35N25		12	2	1	0	−0.546
B36N24	*1-*B36N24	12	3	0	0	−0.459
	*2-*B36N24	18	0	0	0	−0.474

Formation enthalpy is denoted as F.E. The number of B-B, B-B-B, N-N and N-N-N homonuclear bonds is given in each column for each BN-60 cage.

**Table 2:** Adsorption energies of O_2_, CO and CO_2_ molecules on homonuclear boron sites.

Configuration		E_ad_(O_2_) (eV)		E_ad_(CO) (eV)		E_ad_(CO_2_) (eV)	
B25N35		PBE	PBE-D	PBE	PBE-D	PBE	PBE-D
	B1	−0.094	−0.198	0.003	−0.070	0.060	−0.066
	B2	−2.854	−2.988	−0.382	−0.500	−0.195	−0.375
*1*-B27N33	B1	0.001	−0.057	0.003	−0.073	0.038	−0.061
	B2	−2.821	−2.950	−0.539	−0.652	0.052	−0.079
	B3	−2.903	−3.043	−1.185	−1.294	−0.196	−0.382
*1*-B30N30	B1	−0.004	−0.073	0.039	−0.049	0.055	−0.059
	B2	−2.492	−2.622	−0.269	−0.379	0.022	−0.093
	B3	−3.470	−3.599	−1.628	−1.734	−0.858	−1.026
B34N26	B1	−0.001	−0.060	0.022	−0.337	0.048	−0.048
	B2	−2.760	−2.900	0.028	−0.344	0.016	−0.095
	B3	−3.366	−3.495	−1.678	−1.784	−1.214	−1.361

**Table 3:** Bader charges on B1, B2 and B3 sites of *1*-B27N33 before and after adsorption of molecules, in units of *e*.

Configuration	Before adsorption	CO adsorption	O_2_ adsorption	CO_2_ adsorption
B1	2.129	2.146	2.145	2.135
B2	1.344, 1.441	1.423, 1.577	2.19, 2.16	1.319, 1.469
B3	1.25, 0.880, 1.26	1.519, 0.949, 1.411	1.414, 1.378, 2.16	1.405, 1.376, 1.976
