# Supplementary material for: Activation of CO and CO2 on homonuclear boron bonds of fullerene-like BN cages: first principles study
Source: Sci Rep. 2015 Dec 2;5:17460. doi: 10.1038/srep17460 (PMC4667194; doi:10.1038/srep17460)
Supplement: Supplementary Information [file srep17460-s1.pdf]

Supplementary Information

Activation of CO and CO_2 on homonuclear boron bonds of fullerene-like BN cages: first principles study

S. Sinthika[#1], E. Mathan Kumar [#1], V. J. Surya[2], Y. Kawazoe[2,3], Noejung Park[4], K. Iyakutti[*5], Ranjit Thapa[*1,5]

[1]*SRM Research Institute, SRM University, Kattankulathur, Tamil Nadu, 603203, India*
[2]*New Industry Creation Hatchery Center (NICHe), Tohoku University, Sendai, Japan*
[3]Thermophysics Institute, Siberian Branch, Russian Academy of Sciences, Russia
[4]*Center for Multidimensional Carbon Materials, Institute for Basic Science (IBS), Ulsan 689-798, Republic of Korea*
[5]*Department of Physics and Nanotechnology, SRM University, Kattankulathur-603203*

[#]These authors contribute equally to this work
[*]Corresponding Author, Email: ranjit.t@res.srmuniv.ac.in, ranjit.phy@gmail.com (RT); iyakutti@gmail.com (K.I)

Content:

1. Figure S1
2. Figure S2
3. Figure S3
4. Figure S4
5. Figure S5
6. Figure S6
7. Microkinetic Modeling for Sabatier Activity
8. Table S1
9. Table S2

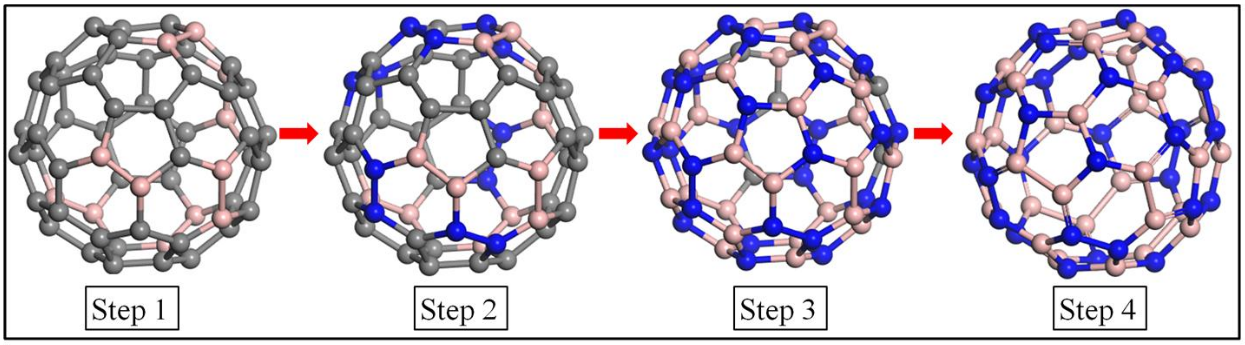

Figure S1. The steps involved for constructing the stoichiometric *1*-B30N30 type cage with 5 B-B, 2 B-B-B & 5 N-N, 2 B-B-B bonds.

Here we provide an understanding about how to make a B30N30 cage. To construct *1*-B30N30 type cage, the first two steps involve replacing C-C bonds in each pentagon by B-B (step 1) and N-N bonds (step 2), so that there are 6 B-B and 6 N-N bonds in the cage. Depending on the distance between B2 and N2 bonds, different types of stoichiometric B30N30 can be obtained. Here out of six pentagons, four B2 bonds are separated by one atom and two B2 bonds are separated by two atoms. The same procedure is followed for the N2 bonds to complete the filling of pentagons. In step 3, we replace the remaining carbon atoms with alternating B and N atoms (C-C by B-N) in maximum possibility. In final step 4, we placed the B or N atom in place

of remaining carbon atoms, while restricting the homonuclear bonds to a maximum of three B (B-B-B) and three N (B-B-N) atoms.

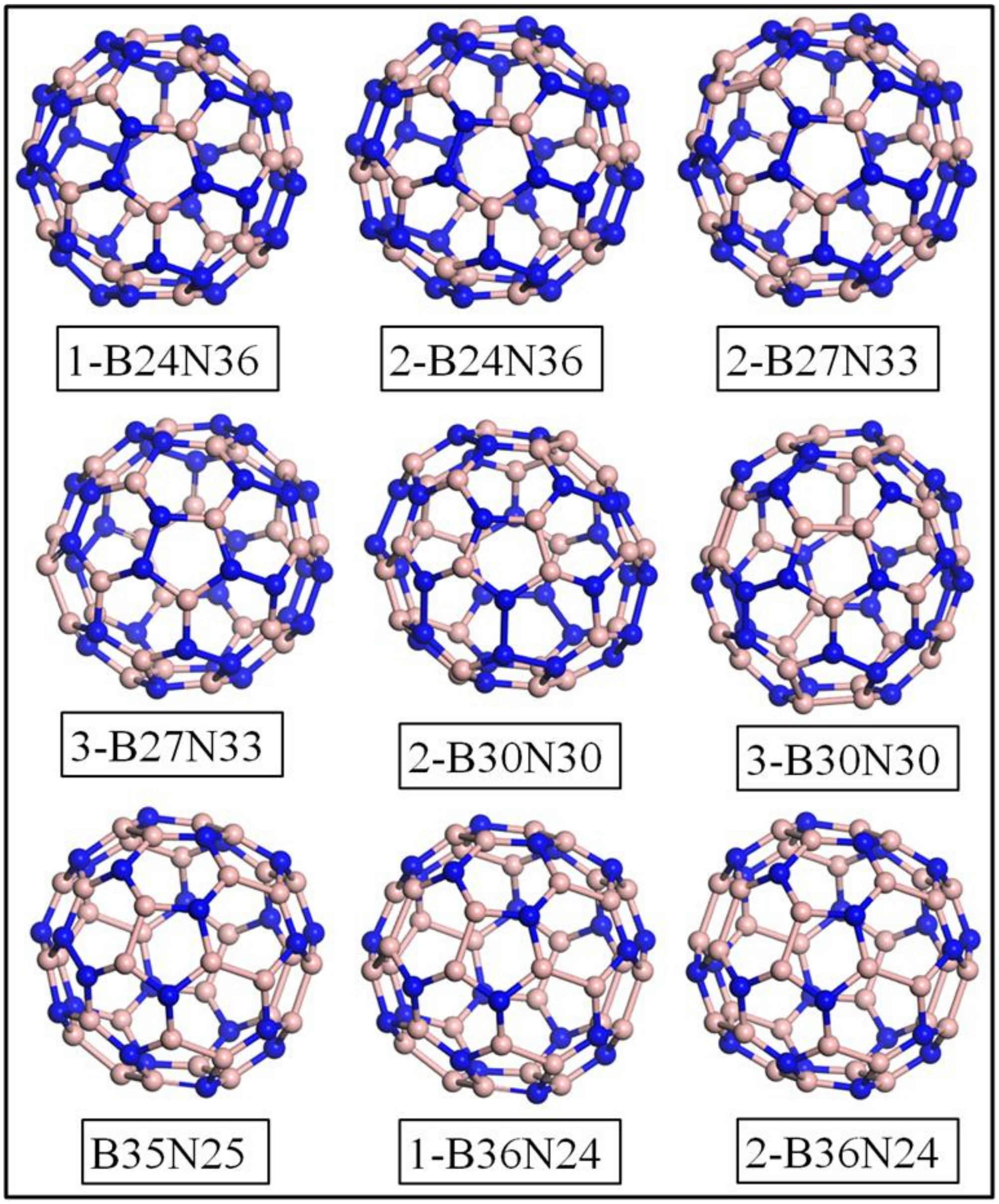

Figure S2. Optimized structure of BN-60 cages with different B:N ratio and homonuclear bonds.

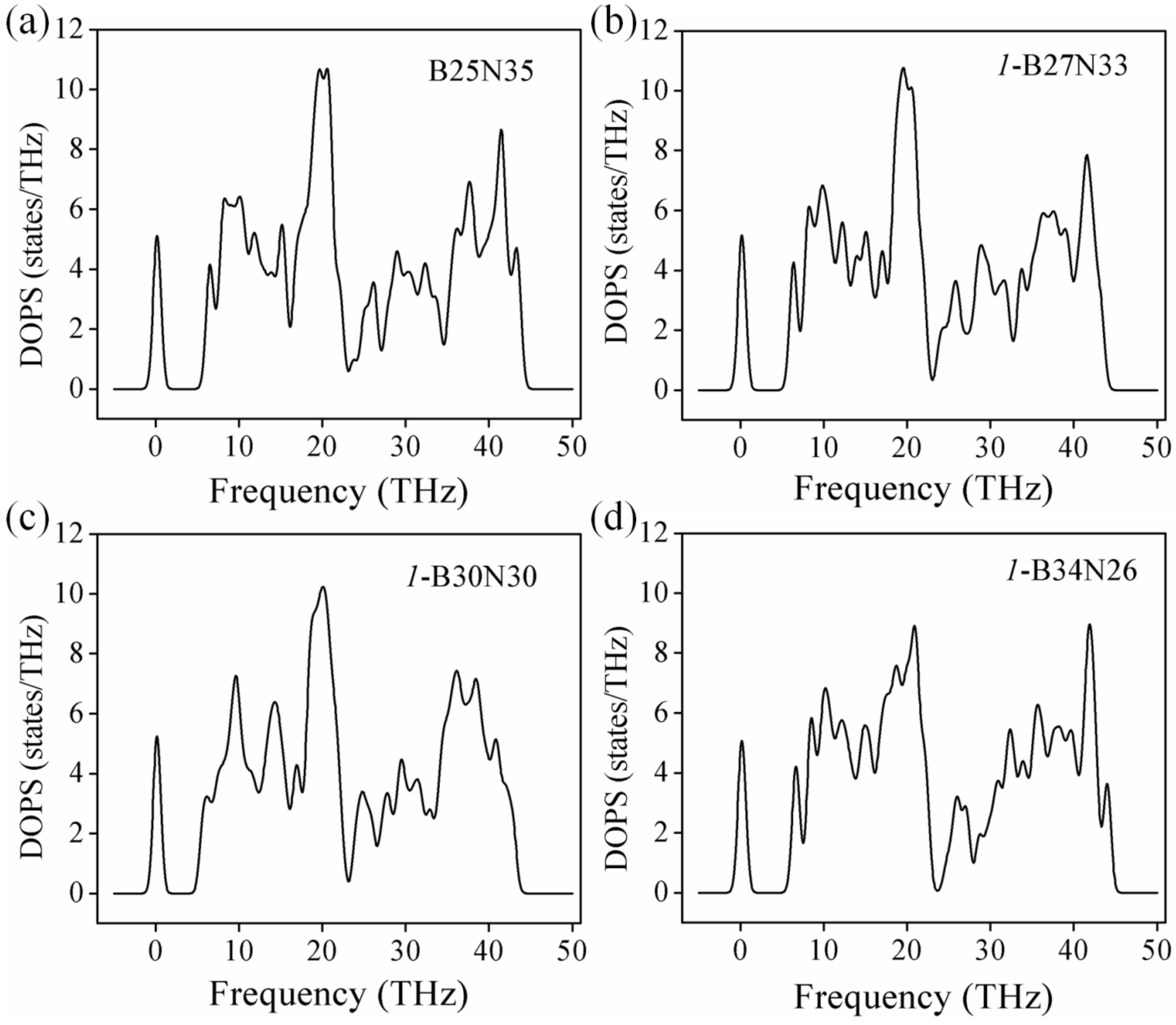

Figure S3. Density of Phonon states of four different BN-60 cages as a function of Frequency (a) B25N35 (b) *1*-B27N33 (c) *1*-B30N30 and (d) *1*-B34N26. As we estimated the DOPS using plane wave based VASP code, for confined system we use 1x1x1 k-points. So at around 0 THz a peak has been observed in all the cases, which is related to translational motion of whole structure. The peak has no relation with the structural instability.

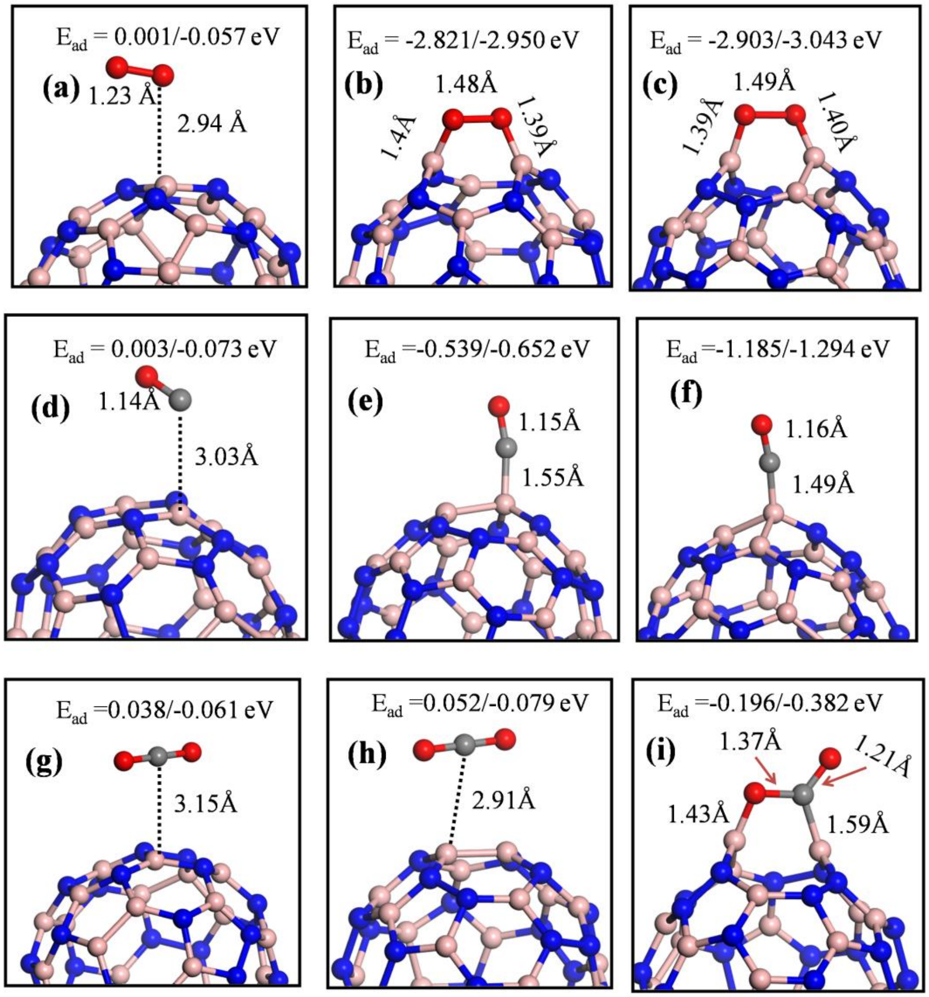

Figure S4.Optimized geometries of adsorption of (a), (b), (c) O_2 molecule on B1, B2, B3 respectively; (d),(e),(f) CO molecule on B1, B2, B3 sites respectively; (g), (h), (i) CO_2 molecule on B1, B2, B3 sites respectively on *1*-B27N33 cage.E_{ad} is denoted for adsorption energy of particular molecule. $E_{ad}$is estimated using PBE/PBE-D functional.

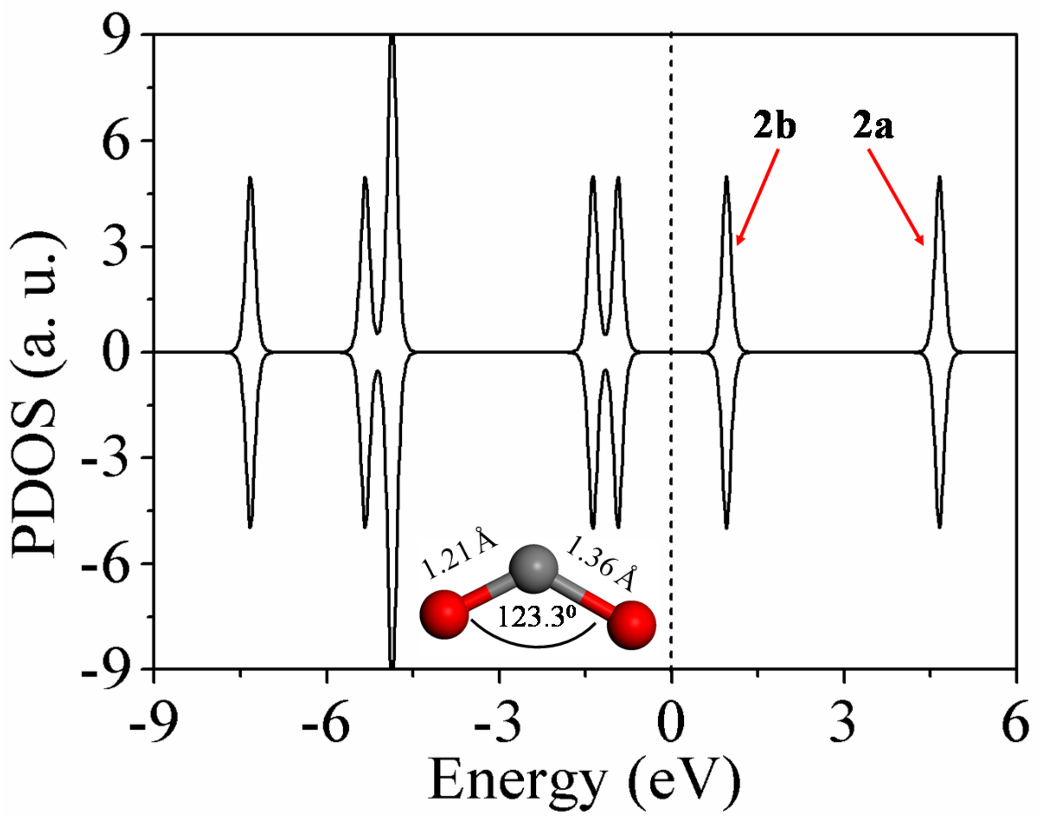

Figure S5: Density of states of CO_2 molecule in the bent configuration. It can be seen that the $2\pi^*$ orbital splits into two non-degenarate levels above the Fermi level.

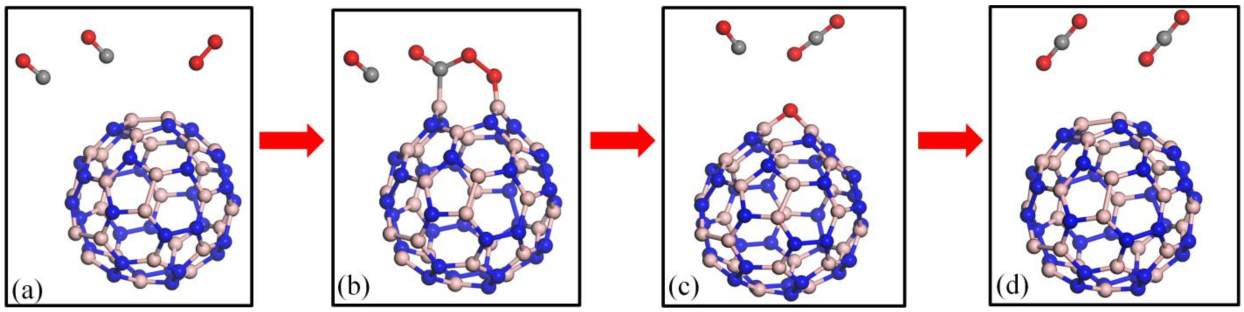

Figure S6. Optimized geometries of CO oxidation reaction steps on B2 site of B27N33.

Microkinetic Modeling for Sabatier Activity:

The Sabatier activity was calculated using the microkinetic model for the LH mechanism as follows:

The reaction proceeds as:

$CO + * \rightarrow CO^*$ (R1)

$O_2 + * \rightarrow O_2^*$ (R2)

$O_2^* + CO^* \leftrightarrow (O_2 \cdots CO)^* \rightarrow CO_2 + O^*$ (R3)

$CO + O^* \rightarrow CO_2 + *$ (R4)

R1 and R2 denote the adsorption of CO and O_2 respectively while in R3, the simultaneous adsorption of the two reactants results in a stable intermediate that reacts to form CO_2.

Reactions R1 and R2 are assumed to be in equilibrium so that the forward and reverse rates are equal:

$$k_1^+ p(CO)\theta_* = k_1^- \theta_{co}$$

$\theta_{co} = K_1 p(CO)\theta_*$ and

$$k_2^+ p(O_2)\theta_* = k_1^- \theta_{o_2}$$

$$\theta_{o_2} = K_2 p(O_2)\theta_*$$

The rate determining step is R3 and hence the rate of CO_2 formation can be taken as the maximum of the rate of the reaction R3. Sabatier activity is the upper bound of the reaction rate R3, considering its forward rate alone:

$$(\text{R3})\text{max} = k_3^+ \theta_{co}\, \theta_{o_2} = k_3^+ K_1 p(CO)\theta_*\, K_2 p(O_2)\theta_*$$

Here,

$$k_3{}^+ = \frac{kT}{h} e^{\frac{-E_a}{kT}}$$

E_a is the activation energy for CO_2 formation.

$K_i$is the equilibrium constant of reaction i,

p(CO) and *p(O₂)* are the partial pressures of CO and O_2: *p(CO)* = 0.01 bar and *p(O₂)* = 0.21 bar at room temperature,

$k_i^+$and$k_i^-$are the forward and backward rate constants for Ri respectively.

In order to calculate the coverages of CO and O_2 in terms of the free sites (θ_*) we employ the rule,

$$\theta_{o_2} + \theta_{co} + \theta_* = 1$$

$$\theta_* = \frac{1}{1 + K_1 p(CO) + K_2 p(O_2)}$$

With the rate constants given by

$$K_1 = e^{\frac{-\Delta G_1}{kT}}$$

where$\Delta G_1 = \Delta E_1 - T\Delta S_1$ is the change in Gibb's free energy of R1

$\Delta E_1$is the adsorption energy of CO

$\Delta S_1$is the change in entropy during CO adsorption

k is the Boltzmann constant

T is the temperature.

$$K_2 = e^{\frac{-\Delta G_2}{kT}}$$

where$\Delta G_2 = \Delta E_2 - T\Delta S_2$ is the change in Gibb's free energy of R2

$\Delta E_2$is the adsorption energy of O_2

$\Delta S_2$is the change in entropy during O_2 adsorption

Finally, the Sabatier activity is calculated as

$$Activity = kT \ln(\frac{(R3)max}{h/kT})$$

Effects of Spin:

The oxygen molecule is initially in the triplet state before interacting with CO and the nanocage. This is confirmed by our VASP results, wherein the magnetic moment of an isolated oxygen molecule is calculated to be $2\mu_B$, indicating a spin multiplicity of 3. The first step of CO oxidation may either be, **case (i)** : the adsorption of CO on the nanocage (CO*) followed by O_2 adsorption, to form an intermediate $(CO\cdots O_2)^*$, or **case(ii)** : the adsorption of molecular oxygen (O_2*) followed by interaction with CO to form CO_2 via ER mechanism. We analyze the change in the magnetic moment of the system in detail for the two cases on B2 and B3 sites of B27N33 cage. In both the cases considered, the triplet O_2 molecule loses its magnetic moment upon adsorption, indicating that it is the first step that involves spin switching of O_2 from triplet to non-magnetic state. The relative energies, (calculated by taking an isolated O_2 molecule in the triplet state far from the nanocage) for each case and on different sites (B2, B3) are tabulated below. It can be observed that the adsorbed O_2 molecule on the nanocage always prefers to have a magnetic moment of zero as is evident from the lower relative energies. The change from triplet to singlet state of O_2 upon adsorption on the nanocage is also evident from the density of states shown in figure 5b and 5c of the main manuscript. The $2\pi^*$ state of the O_2 molecule is occupied because of electron donation from the nanocage. The oxygen molecule hence loses its magnetic moment and converts to a singlet state.

Case	System (B27N33)	*Magnetic moment, m (μ_B)	Relative energy PBE/PBE+D (eV)
(i)	Far O_2	2	0
	O_2 adsorbed at B2	0	-2.11/-2.22
		2	-0.04/-0.15
	O_2 adsorbed at B3	0	-2.03/-2.13
		2	-0.51/-0.62
(ii)	Far O_2	2	0
	O_2 adsorbed at B2	0	-2.82/-2.95
		2	-0.64/ -0.76
	O_2 adsorbed at B3	0	-2.90/-3.05
		2	-0.51/-0.65

Table S1. Demonstrates the relative total energy of absorbed system with two different magnetic moment compared to the system with O_2 in triplet state far from the surfaces (taken as reference). The more –ve energy indicate the lower total energy and hence better stability in that state. * The initial magnetic moment of O_2 was assigned to be m (either 0 or 2) along with the constraint that $N_\uparrow$- $N_\downarrow$ = m, where $N_\uparrow$ is the total number of up spin electrons in the system and $N_\downarrow$ is the total number of down spin electrons in the system, as calculated in previous works employing plane wave basis-set codes[1].

In comparison with the conventional catalyst, gold and silver nanoparticles have proven to be very efficient in the catalytic conversion of CO to CO_2. Comparison of Sabatier activities of these metal nanoparticle-based catalysts with the proposed BN-60 cages and Stone-Wales BN nanotube (SW-BNNT) would provide a qualitative estimate of the efficiency of the metal free systems. The Sabatier activities (S.A) of a few selected catalysts are tabulated below

System	E_{ad} (O_2) (eV)	E_{ad} (CO) eV	Sabatier Activity
**13Ag-*(Icosahedron)*[(2)]**	-0.65	-0.88	-0.23
**12Ag-1Pd*(Icosahedron)* [(2)]**	-0.7	-0.57	-0.65
**12Ag-1Ni*(Icosahedron)* [(2)]**	-0.62	-0.5	-1.05
**12Ag-(two layered cluster)[(2)]**	-1.0	-0.37	-1.1
**12Au-(two layered cluster)[(3)]**	-0.5	-0.9	-0.6
**12Pt-(two layered cluster)[(3)]**	-2.75	-2.3	-1.4
**Pt(111)[*(4)]**	-1.0	-1.25	-1.0
**Pd(111)[*(4)]**	-1.2	-0.9	-1.0
**Rh(111)[*(4)]**	-1.8	-1.6	-1.4
**Pt55[(5)]**	-1.79	-1.51	-0.24
**Au55[(5)]**	-0.49	-0.77	0.48
	**Present Work**		
**B25N35 (at B2 site)**	-2.98	-0.5	-1.8
**B27N33 (at B2 site)**	-2.95	-0.65	-1.3
**B30N30 (at B2 site)**	-2.62	-0.38	-0.61
**SW-BNNT**	-2.97	-0.08	-2.60

*The S.A were calculated at T=600 K, p(O_2)=0.33 bar, and p(CO)=0.67 bar, for all other cases, it was calculated at low temperature conditions (T=273 K, p(O_2)=0.21 bar, and p(CO)=0.01)

Table S2: Demonstrates the O_2 and CO adsorption energy on the various catalysts and Sabatier activities of the catalysts.

On comparing the S.A of the nanocages with that of other catalysts, it can be observed that the proposed BN-60 nanocages may exhibit catalytic activity comparable to a few other conventional catalysts and the B30N30 cages have SA that is almost equal to 12Ag-1Pd and 12Au. Also, in order to develop future metal-free catalysts based on these BN nanocages, the

boron-boron bonds have to be tailored effectively so that the reactivity of the system toward CO oxidation is enhanced, while at the same time not compromising the stability.

References:

1. Fan, X. L., Zhang, Y. F., Lau, W. M. & Liu, Z. F. Adsorption of Triplet O_2 on Si(100): The Crucial Step in the Initial Oxidation of a Silicon Surface. *Phys. Rev. Lett.* **94**, 016101 (2005).
2. Kim, H. Y., Kim, D. H., Ji Hoon Ryu and Hyuck Mo Lee. Design of Robust and Reactive Nanoparticles with Atomic Precision: 13Ag-Ih and 12Ag-1X (X = Pd, Pt, Au, Ni, or Cu) Core-Shell Nanoparticles. *J. Phys. Chem. C*, **113**, 15559–15564 (2009).
3. Falsig, H., Hvolbæk, B., Kristensen, I. S., Jiang, T., Bligaard, T., Christensen, C. H. & Nørskov, J. K. Trends in the Catalytic CO Oxidation Activity of Nanoparticles. *Angew. Chem.* **120**, 4913-4917 (2008).
4. Nørskov, J. K., Bligaard, T., Hvolbæk, T., Pedersen, F. A., Chorkendorffc, IB. & Christensen, C. H. The nature of the active site in heterogeneous metal catalysis. *Chem. Soc. Rev*. **37**, 2163-2171 (2008).
5. Sinthika, S., Kumar, E. M. & Thapa, R. Doped h-BN monolayer as efficient noble metal-free catalysts for CO oxidation: the role of dopant and water in activity and catalytic de-poisoning. *J. Mater. Chem. A.* **2**, 12812-12820 (2014).
